# Experiences and perceptions of patients with cancer receiving home-based chemotherapy: a qualitative systematic review protocol

**DOI:** 10.1186/s13643-024-02659-1

**Published:** 2024-09-28

**Authors:** Porawan Witwaranukool, Ratchanok Phonyiam, Yanni Wu, kathryn Kynoch

**Affiliations:** 1 Ramathibodi School of Nursing, Faculty of Medicine Ramathibodi Hospital, Mahidol University, Bangkok, Thailand; 2 Mahidol University Ramathibodi School of Nursing: A JBI Affiliated Group, The University of Adelaide, Adelaide, Australia; 3 Nanfang Hospital, Southern Medical University, Guangdong, China; 4 The Nanfang Nursing Centre for Evidence-based Practice: A JBI Centre of Excellence, Adelaide, Australia; 5 Mater Health and Queensland Centre for Evidence-Based Nursing and Midwifery: A JBI Centre of Excellence, Brisbane, Australia; 6 School of Nursing, Queensland University of Technology, Kelvin Grove, Brisbane, Australia

**Keywords:** Experiences, Chemotherapy, Cancer, Home

## Abstract

**Background:**

Home-based chemotherapy (HBC) has emerged as a standard option for treating various types of cancer, primarily to decrease the waiting time for treatment. As HBC gains more recognition, ongoing research is delving into the experiences of patients with cancer who receive chemotherapy in a home setting or chemotherapy closer to home. Understanding these experiences is vital for the use of chemotherapy delivery outside the traditional hospital environments. This review aims to synthesize and critically appraise qualitative studies that investigate the experience and perspectives of patients with cancer who received parenteral chemotherapy administration in home settings. Findings will be used to develop evidence-based policies to support home-based care models.

**Methods:**

This review will follow JBI methods for systematic reviews of qualitative evidence. The databases for searching will include MEDLINE (PubMed), CINAHL (EBSCOhost), PsycINFO (EBSCOhost), ProQuest Health and Medical Collection, two Chinese databases, CNKI and Wanfang, and one Thai database, ThaiJO. Studies published in English, Chinese, and Thai will be considered for inclusion. Two reviewers will independently undertake study selection, data extraction, and critical appraisal of the methodological quality of studies. The synthesized findings will be assessed using the ConQual approach.

**Discussion:**

The synthesis of qualitative studies on this topic will provide insights into the nuanced and varied experiences of patients receiving chemotherapy within the comfort of their homes. The review will also provide evidence-based recommendations to policymakers and healthcare administrators, to support the implementation of HBC for patients.

**Systematic review registration:**

Systematic review registration: PROSPERO CRD42024500476.

**Supplementary Information:**

The online version contains supplementary material available at 10.1186/s13643-024-02659-1.

## Introduction

Cancer is one of the leading causes of morbidity and mortality worldwide, with millions of people affected each year [1]. The journey of cancer treatment is complex and multifaceted, involving various modalities such as surgery, radiation, and chemotherapy [2]. Of these, chemotherapy remains a cornerstone, playing a crucial role in both curative and palliative scenarios for many types of cancer [2]. This treatment is typically administered in clinical settings, such as inpatient wards and ambulatory units, due to the need for precise dosage control, management of side effects, patient safety, and the requirement for a sterile, controlled environment [3]. Adherence to chemotherapy to control cancer-related symptoms is an important aspect of treatment for almost all cancer patients [4]. However, previous research has highlighted the importance of the emotional and practical support of families and friends in meeting patients' needs during this time [4].

Rising cancer rates, evolving treatment methods, and earlier diagnosis present various important challenges and opportunities for both healthcare professionals and patients, and therefore, home-based chemotherapy is increasing as a treatment option to address these issues [5]. Over the past two decades, administering intravenous chemotherapy at home or home-based chemotherapy (HBC) has become the standard treatment for many types of cancers [5–7]. HBC allows patients with cancer to receive their cancer treatment in the comfort of their own homes, and thus, this is in line with wide healthcare trends that focus on patient autonomy, personalized care, and the shift away from institutionalized treatment settings [6]. Moreover, HBC has seen an increased demand for healthcare services that are centered around the patients’ needs and preferences [6], reflecting a broader movement in healthcare to make treatments less burdensome and more integrated into patients' daily lives. Additionally, HBC can be particularly beneficial for patients undergoing long-term treatments like chemotherapy, where the comfort and familiarity of the home environment can play a significant role in their overall well-being [5].

The COVID-19 pandemic significantly impacted cancer care, leading to delays and discontinuations in treatments as well as necessitating changes to chemotherapy protocols [7]. This situation has heightened concerns about the effectiveness of cancer therapies, exacerbating anxiety and distress among patients and their families [4]. Many patients with cancer are worried about the anticipated outcomes of their treatment and its impact on their quality of life [8]. In response, there has been a significant increase in the use of HBC as a safer and more practical alternative [8]. HBC enables patients to receive essential treatment while minimizing their risk of exposure to various infections, including COVID-19. The transition to HBC aids in maintaining patient treatment schedules and reduces the risk of exposure to infections for these vulnerable patients. It also reflects the evolving nature of healthcare delivery, highlighting how home-based treatments have become integral in managing complex conditions such as cancer in a home setting [9]. As a result, there has been a noticeable shift toward HBC for various types of cancer, marking a significant change in the approach to cancer treatment during these challenging times [10].

Previous qualitative studies focusing on cancer patients’ experiences with HBC have highlighted many benefits as well as challenges with delivering these types of home-based treatments [11–14]. One of these studies reported that patients with cancer felt more at ease when receiving chemotherapy at home and they felt less sick and more able to handle treatments [5]. Additionally, HBC helps to alleviate the psychological stress often associated with clinical settings, with studies highlighting that patients report they enjoy receiving treatment in the comfort of their own familiar environment, particularly for patients with mobility issues or those living in rural areas [12]. However, the transition to HBC presents numerous challenges. It requires a robust healthcare infrastructure capable of supporting home care, including skilled nursing staff, efficient logistics for drug delivery, and effective communication systems [6]. Patients’ education becomes even more critical, as they need to be well-informed about drug administration, side effect management, and emergency procedures [15]. Experiences of patients with cancer can vary widely based on individual circumstances, including the type and stage of cancer, the specific chemotherapy regimen, and the patient’s overall health and support system [3, 11]. Understanding the experiences of patients with cancer receiving HBC is essential to optimizing the care they receive and maximizing the advantages of this treatment approach.

A preliminary search of PROSPERO, MEDLINE, the Cochrane Database of Systematic Reviews, and JBI Evidence Synthesis was conducted to identify existing systematic reviews on this topic. A systematic review by Corbett et al. [16] from 2013 found that the review included both quantitative and qualitative studies and primarily focused on the economic aspects of administering intravenous chemotherapy outside of traditional hospital settings. Their review did not explore the experiences and perceptions of cancer patients undergoing chemotherapy at home. In addition, given the timeframe since the review by Corbett and colleagues was published, an updated review is needed to incorporate more recently published qualitative research on this topic [13, 14, 17, 18]. Therefore, this review aims to explore cancer patients’ experiences and perceptions while undergoing chemotherapy at home. The review will seek to provide a comprehensive understanding of how patients manage and perform self-care for intravenous chemotherapy at home, including the barriers, challenges, and benefits of home-based chemotherapy. The findings will assist healthcare professionals in delivering personalized care that addresses patients’ physical and emotional needs. In addition, the evidence-based recommendations will inform local policies and procedures to support the implementation of home-based chemotherapy.

## Objectives

The main objective of this systematic review is to synthesize qualitative studies investigating the experiences and perceptions of patients with cancer receiving home-based chemotherapy, including the barriers, challenges, and benefits of treatment. This review will consider the following questions:

1. What are the experiences and perceptions of patients with cancer in receiving chemotherapy in their home environment?
2. What are the barriers, challenges, and benefits identified by patients receiving home-based chemotherapy?

## Methods

This review protocol has been registered in the PROSPERO database under the registration number CRD42024500476. The proposed systematic review will be conducted in accordance with the (Joanna Briggs Institute) JBI methodology for systematic reviews of qualitative evidence [19]. This review protocol is being reported in accordance with the reporting guidance provided in the Preferred Reporting Items for Systematic Reviews and Meta-Analyses Protocols (PRISMA-P) statement [20] (see checklist in Additional file 1).

### Study eligibility criteria

The eligibility criteria for this review are based on the JBI methodology for systematic reviews of qualitative evidence [19]. These criteria encompass participants, phenomena of interest, context, and types of studies (see Table 1). Studies that include adult patients (over 18 years) with cancer receiving home-based chemotherapy will be considered for inclusion [21]. The review will include studies involving patients with any type of cancer and at any phase of chemotherapy, including those who have completed HBC. Cancer patients who never received HBC will be excluded. The phenomena of interest for this review are the experiences and perceptions of cancer patients receiving home-based chemotherapy. Any aspect of treatment will be included, such as the administration of intravenous chemotherapy at home, self-management during cancer treatment, and other related aspects of treatment. Studies that include patients receiving oral anticancer medications will be excluded, as these treatments do not traditionally require hospital-based care, and patients typically self-administer these medications. The context will include studies conducted in the home environments of cancer patients, where “home” refers to the patient’s residence or any non-hospital setting where chemotherapy is administered [21]. This setting can include various safety and monitoring measures, such as nurse visits, telehealth check-ins, and specialized equipment for drug administration. Lastly, this review will focus on studies that provide qualitative data, covering research designs such as phenomenology, grounded theory, ethnography, action research, and feminist research as well as mixed methods papers and other qualitative designs where qualitative findings can be separately extracted. No specific timeframe limit will be applied to the search for papers, allowing for the inclusion of all studies on this topic, including those during the COVID-19 pandemic, where an increase in the uptake of HBC has been observed.

**Table 1:** Application of eligibility criteria of the current review

Criterion	The current review	
	Inclusion criteria	Exclusion criteria
Participants	Age above 18 years, with any type of cancer at any stage, without restrictions based on gender, ethnicity, or socioeconomic status	No HBC involvement
Phenomena of interest	Experiences and perceptions of patients with cancer receiving HBC through parenteral anticancer treatment	Oral anticancer medications
Context	Home settings include patient’s residence or other long-term living environments	Traditional cancer settings, including inpatient care units and ambulatory units
Types of studies	Qualitative research and qualitative data collection methods	Studies that used survey data or statistical reporting of results, commentaries, or discussions on the subject

### Search strategy

The search strategy will focus on identifying both published and unpublished research. This review will employ a three-stage search strategy. Firstly, an initial limited search of MEDLINE (PubMed) and CINAHL (EBSCO) was undertaken to identify articles on the topic. The text words contained in the titles and abstracts of relevant articles, and the index terms used to describe the articles were used to develop a full search strategy for MEDLINE (PubMed) (see Appendix 1). Secondly, the search strategy will be modified for each included information source, and a subsequent search will be conducted across all selected databases, including MEDLINE (PubMed), CINAHL (EBSCOhost), PsycINFO (EBSCOhost), ProQuest Health and Medical Collection, two Chinese databases, CNKI and Wanfang and one Thai database, ThaiJO. In addition to searching the published literature, we will also explore sources of unpublished studies and gray literature for additional papers. This will include searching Google Scholar, OpenGrey, and ProQuest Dissertations and Theses Global. Thirdly, the reference list of all included studies will be screened to identify further papers for inclusion. Studies published in English, Chinese, and Thai will be included.

### Study selection

Following the search, all identified citations will be compiled and uploaded into EndNote version 21 (Clarivate Analytics, PA, USA). Following the removal of duplicates, titles, and abstracts will then be screened by two independent reviewers for assessment against the inclusion and exclusion criteria for the review. Potentially relevant studies will be retrieved in full and their citation details imported into the JBI System for the Unified Management, Assessment, and Review of Information (JBI SUMARI) (JBI, Adelaide, Australia) [22]. The full text of selected citations will be assessed in detail against the inclusion criteria by two independent reviewers. Reasons for exclusion of papers in full text that do not meet the inclusion criteria will be recorded and reported in the systematic review. Any disagreements that arise between the reviewers at each stage of the selection process will be resolved through discussion, or with a third reviewer. The results of the search and the study inclusion process will be reported in full in the final systematic review and presented in a Preferred Reporting Items for Systematic Reviews and Meta-analyses (PRISMA) flow diagram [20].

### Assessment of methodological quality

Eligible studies will be critically appraised by two independent reviewers for methodological quality using the standard JBI Critical Appraisal Checklist for Qualitative Research [19]. This 10-item tool assesses the methodological quality of a study across various aspects, including the formulation of research questions, the chosen methodology, the interpretation of results, and ethical considerations, using response options of “yes,” “no,” “unclear,” or “not applicable.” Authors of papers will be contacted to provide any missing or supplementary data necessary for clarification. In cases where discrepancies occur among the reviewers, these will be resolved through discussion or by consulting a third reviewer. The findings from the quality appraisal will be presented both in a narrative format and using tables in the final systematic review report.

### Data extraction

Data will be extracted from studies included in the review by two independent reviewers using the standardized JBI data extraction tool in JBI SUMARI [19]. The data extracted will include specific details about the population, context, culture, geographical location, study methods, and the phenomena of interest, the experiences, and perceptions of patients with cancer receiving chemotherapy at home. Findings and their associated illustrations will be extracted verbatim and assessed for credibility using the JBI Levels of Credibility. These levels, based on the reviewers' judgment of how well each illustration represents its related finding, are then sorted into one of three categories: Unequivocal (indicating that the finding is supported by an illustration that is indisputable and not subject to challenge), Credible (indicating that the finding is supported by an illustration with a weak or unclear connection, making it open to challenge), or not supported (indicating that the data do not back the finding) [19]. Any disagreements that arise between the reviewers will be resolved through discussion or with a third reviewer. Authors of papers will be contacted to request missing or additional data, where required.

### Data synthesis

Where possible, qualitative research findings will be combined using the JBI SUMARI tool using a meta-aggregation approach [22]. Meta-aggregation involves grouping or synthesizing findings to create a set of statements that reflect this aggregation by organizing the findings and categorizing them based on their similarity in meaning. These categories are then synthesized to produce a single, comprehensive set of findings that can serve as a foundation for evidence-based practice. Only findings that are unequivocal and credible will be included in the aggregation, while unsupported findings will be presented separately. Findings will be presented in a meta-aggregation flow chat or table as well as a narrative summary.

### Assessing confidence in the findings

The final synthesized findings of this review will be evaluated and graded using the Confidence in Qualitative Synthesis Findings (ConQual) approach, a well-established method for assessing confidence in qualitative research synthesis [23]. This evaluation will be presented in a comprehensive “Summary of findings” section. The summary of the findings will provide a concise overview of the review, including the title, target population, phenomena of interest, and the overall research context. Each synthesized finding will be presented along with the following key details:

- Type of research: this specifies the particular qualitative research method that contributed to the finding (e.g., interview, focus group, and ethnography).
- Dependability score: this shows the degree of certainty regarding the finding's precision and consistency, evaluated using the ConQual criteria.
- Credibility score: this represents the confidence level in the reliability and genuineness of the finding, also determined through the ConQual framework.
- Overall ConQual score: this consolidates the dependability and credibility ratings into a unified metric, providing an overall measure of confidence in the integrated finding.

By presenting the findings in this transparent and structured manner, we aim to provide a clear and reliable basis for interpreting and drawing conclusions from the review. This will enable healthcare professionals and policymakers to make informed decisions about HBCs based on the most robust and credible evidence available.

## Discussion

This proposed systematic review of qualitative evidence represents a pioneering effort to synthesize the experiences and perceptions of cancer patients undergoing HBC. The synthesis of qualitative studies will provide insights into the nuanced and varied experiences of patients receiving chemotherapy within the comfort of their homes. These insights are invaluable, illuminating the impact of HBC on patients' quality of life, autonomy, and emotional well-being. By exploring this evidence, we aim to identify the benefits, barriers, and challenges of this mode of treatment. The push toward the adoption of HBC, spurred by the constraints imposed by the COVID-19 pandemic on traditional healthcare delivery, has made it crucial to understand its impact from the patients’ perspective. Patients’ experiences and perspectives highlighted in this review will provide important aspects related to patient experience and how patients’ needs can be met to mitigate the emotional and psychological burdens associated with chemotherapy.

The recent shift toward home-based care models is emblematic of a broader transformation in oncology practices, emphasizing the need for healthcare systems to adapt to patients’ evolving preferences and circumstances [7]. However, while HBC’s benefits and potential drawbacks are compelling, this review will highlight the challenges and barriers to implementation and wider adoption [3, 11, 15, 17]. The variability in patients’ experiences, influenced by factors such as the type and stage of cancer, underscores the complexity of delivering personalized care at home [3, 11]. The synthesis of qualitative findings will provide a solid foundation for future research, policy development, and clinical practice in HBC. It will highlight the importance of patient education on drug administration, side effect management, and emergency procedures, which are pivotal for the success of home-based treatment models. Additionally, the review will call for ongoing evaluation of HBC’s clinical effectiveness, safety, and patient satisfaction to refine and expand its implementation.

## Supplementary Information

Additional file 1. PRISMA-P 2015 checklist.

## Data Availability

Not applicable.

## Appendix 1: Search strategy

**MEDLINE (PubMed)**

Search conducted on January 25, 2024

**Table:**

Search	Query	Records reviewed
#1	Search: (((experience* [ Title/Abstract]) OR percep*[ Title/Abstract]) OR perceive*[Title/Abstract]) AND [All Fields]	1,905,061
#2	Search: ((((((((qualitative* [MeSH]) OR interview* [ MeSH]) OR “focus group” [MeSH]) OR “mixed method*” [ MeSH]) OR qualitative* [Title/Abstract]) OR interview*[Title/Abstract]) OR narrative*[Title/Abstract]) OR “mixed method*” [Title/Abstract]) AND [All Fields]	843,305
#3	Search: ((((((cancer*[Title/Abstract]) OR neoplas*[Title/Abstract]) OR tumor*[Title/Abstract]) OR tumour* [Title/Abstract]) OR malignan*[Title/Abstract]) OR oncolog*[Title/Abstract]) OR carcinoma*[Title/Abstract] AND [All Fields]	4,170,714
#4	Search: (((((((((((“drug therapy” [MeSH]) OR “antineoplastic combined chemotherapy” [MeSH]) OR chemotherap* [MeSH]) OR “systemic therapy” [MeSH]) OR “adjuvant therapy” [MeSH]) OR chemotherap* [Title/Abstract]) OR “adjuvant therapy” [Title/Abstract]) OR “systemic therapy” [Title/Abstract]) AND [All Fields]	1,882,538
#5	Search: ((((((((((((((“home environment” [Title/Abstract]) OR home [Title/Abstract]) OR communit* [Title/Abstract]) OR "nursing homes" [MeSH]) OR “home environment” [MeSH]) OR home [MeSH]) OR “hospital at home” [MeSH]) or “hospital in the home” [MeSH]) or “own home*” [MeSH]) or “home care” [MeSH]) or “closer to home” [MeSH]) OR communit* [MeSH]) OR "nursing homes" [MeSH]) OR "residence characteristics"[MeSH]) AND [All Fields]	1,338,891
#6	Search: #1 AND #2 AND #3 AND #4 AND #5	247

No limits applied

## Abbreviations

HBC: Home-based chemotherapy
JBI: Joanna Briggs Institute
SUMARI: System for the Unified Management, Assessment and Review of Information
PRISMA: Preferred Reporting Items for Systematic Reviews and Meta-analyses
ConQual: Confidence in qualitative synthesis findings
